# Hypertension in patients admitted to clinical units at university hospital: post-discharge evaluation rated by telephone

**DOI:** 10.1590/S1679-45082017AO3862

**Published:** 2017

**Authors:** Cássia Lima de Campos, Angela Maria Geraldo Pierin, Natalia Alencar de Pinho

**Affiliations:** 1 Hospital Sírio Libanês, São Paulo, SP, Brazil.; 2 Universidade de São Paulo, São Paulo, SP, Brazil.; 3 Centre d’Epidémiologie et Santé des Populations, Institut National de la Santé et de la Recherche Médicale Unité 1018, Villejuif, France.

**Keywords:** Hypertension/prevention & control, Health knowledge, attitudes, practice

## Abstract

**Objective:**

To characterize hypertensive patients after admission to hospital considering the current status, compliance to treatment, habits and lifestyle, and knowledge and beliefs about the disease.

**Methods:**

This was an exploratory study with 265 hypertensive patients admitted to a medical inpatients unit of a university hospital. Data were collected in an interview over the telephone. The level of significance was set as p<0.05.

**Results:**

It was found that 32% of hypertensive patients had died. One hundred patients were interviewed, mean age of 64.15 (13.2) years, 51% were women, 56% non-white, 51% with primary education, 52% were retired, 13% were smokers, 38% used alcohol, 80% did not perform physical exercise, and the mean body mass index was 35.9 (15.5) kg/m^2^. The comorbidities were heart problem (52%), diabetes (49%) and stroke (25%). As to antihypertensive treatment, 75% were on use, 17.3% stopped taking them and 21.3% missed visits. The treatment sites were the primary care unit (49%) and hospital (36%). As for knowledge and beliefs, 25% believed hypertension is curable, 77% that treatment should last for the rest of their lives, and hypertension brings complications (84%). A total of 46.7% were controlled. The lack of control was associated (p<0.05) with non-white ethnicity and absence of heart problems.

**Conclusion:**

There were significant deaths occurred after hospitalization and poor control of blood pressure, probably due to inadequate habits and lifestyles and non-compliance to antihypertensive treatment.

## RESUMO

**Objetivo:**

Caracterizar hipertensos após internação quanto a condição atual, adesão ao tratamento, hábitos e estilos de vida, e conhecimento e crenças sobre a doença.

**Métodos:**

Estudo exploratório, com 265 hipertensos, após internação em clínica médica de hospital universitário. Os dados foram coletados em entrevista por contato telefônico. O nível de significância foi de p<0,05.

**Resultados:**

Verificou-se que 32% dos pacientes faleceram. Foram entrevistados 100 hipertensos, com média de idade de 64,15 (13,2) anos, 51% eram mulheres, 56% não brancos, 51% com 1^o^ grau de escolaridade, 52% eram aposentados, 13% tabagistas, 38% usavam bebida alcoólica, 80% não realizavam exercícios físicos e o índice de massa corporal médio foi de 35,9 (15,5) kg/m^2^_._ As comorbidades foram problema cardíaco (52%), diabetes (49%) e acidente vascular encefálico (25%). Quanto ao tratamento anti-hipertensivo, 75% estavam em uso de medicamentos, 17,3% deixaram de tomá-los e 21,3% faltaram às consultas. O tratamento era feito em unidade básica de saúde (49%) e no hospital (36%). Quanto aos conhecimentos e crenças, 25% acreditavam que hipertensão tinha cura, 77% que o tratamento deveria ser por toda a vida e 84% que a hipertensão trazia complicações. Estavam controlados 46,7% hipertensos. A ausência de controle associou se com etnia não branca e ausência de problemas cardíacos (p<0,05).

**Conclusão:**

Foram expressivas as mortes ocorridas após internação e controle insatisfatório da pressão arterial, provavelmente decorrentes de hábitos e estilos de vida inadequados e não realização adequada do tratamento anti-hipertensivo.

## INTRODUCTION

Arterial hypertension is recognized as one the major public health problems,^1^ representing one of the main risk factors for cardiovascular diseases. According to the Surveillance of Risk and Protection Factors for Chronic Diseases by Telephone Survey (VIGITEL), the frequency of adults that reported a medical diagnosis of arterial hypertension was 24.8%, in 2014.^2^

Data from the American Heart Association show that 40.6% of mortality due to cardiovascular diseases is related to an increase in blood pressure, with hypertension present in 69% of patients in the first episode of acute myocardial infarct, 77% of those with stroke, 75% with heart failure, and 60% with peripheral arterial disease.^3^

For the year 2050, the estimate is for double the number of cases of coronary disease, stroke and hypertension, achieving 34 thousand cases per 100 thousand inhabitants.^4^

Besides the elevated prevalence of hypertension, we point out the unsatisfactory control of hypertensive patients diagnosed. A national review study showed a great variation in the rate of control, varying from 10.0% in Southern microregions, to 57.6% in a multicenter study in cities.^5^ The absence of control of the disease predisposes towards complications. In a study performed in Brazil, which evaluated mortality due to stroke, hypertension was the basic cause of death;^6^ and, in an international study, the complications resulting from hypertension were responsible for 9.4 million deaths a year.^7^

One of the mechanisms already used to evaluate control and compliance, as well as the involvement of organs, was the telephone survey, used nationally by means of the VIGITEL program to follow up hypertensive patients.^2,8,9^

Considering what was exposed, we question which are the events that take place after discharge of hypertensive patients, *i.e.*, after their hospital stay.

## OBJECTIVE

To characterize the follow-up of hypertensive patients relative to antihypertensive treatment after hospital discharge; to identify drug and non-drug antihypertensive treatment; to identify life habits and health styles; to identify the degree of knowledge about aspects related to hypertension and the treatments used; and to evaluate compliance with antihypertensive treatment.

## METHODS

An exploratory-descriptive study was conducted, approved by the Research Ethics Committee, with official opinion no. 74378, CAAE: 04130112.1.0000.5392.

**Figure 1:**
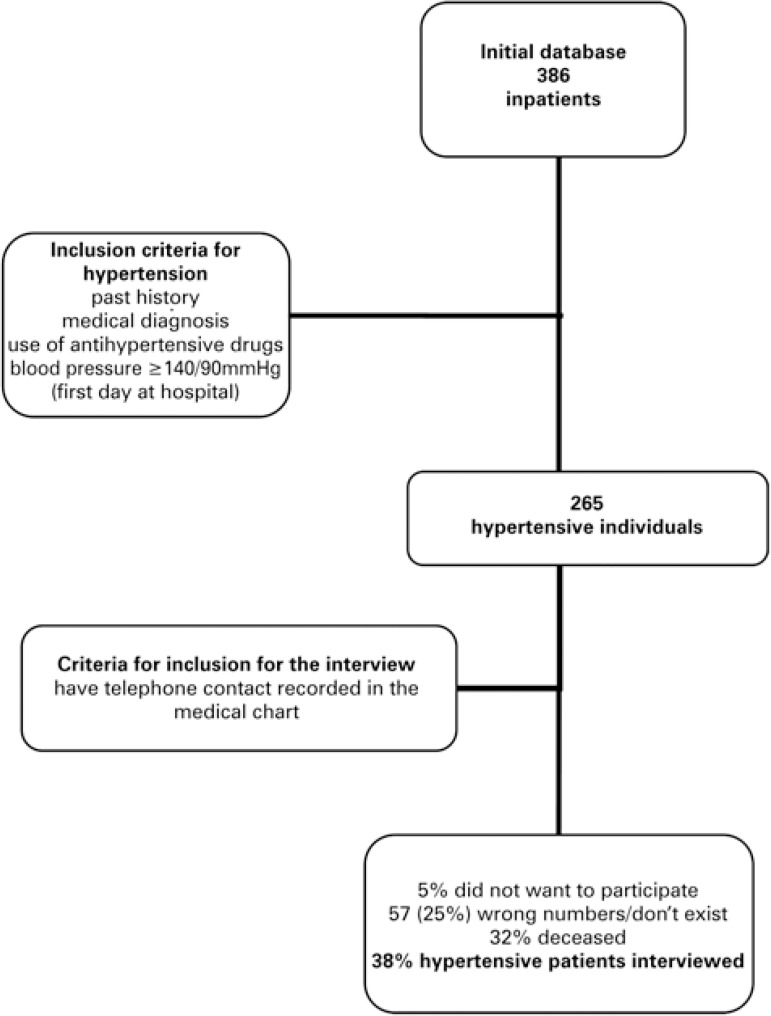
Flowchart of inclusion of hypertensive patients in the study

The population was obtained from the database of a study,^10^ which included adults admitted to the internal medicine inpatients unit of a university hospital in the city of São Paulo (SP), during the period from January 1, 2009 to December 31, 2009. The sample was calculated taking into consideration the estimate of prevalence of a 13% alteration of renal function, 5% variation, 5% type I error, 80% test power. With these parameters, the sample should have been made up of 386 patients (Figure 1).

Within the sample, patients were selected by at least one of four possibilities: positive past history, medical diagnosis, use of antihypertensive drugs, blood pressure ≥140/90mmHg checked on the morning of the first day of hospitalization; 265 adult patients were hypertensive.

All patients that had records of a telephone contact in their medical charts were potential participants. Three attempts were made to locate them, and the data were collected by means of a telephone interview during the period from November 2012 to May 2013. The follow-up of health data by telephone contact is a mechanism that has been used frequently.^2,8^

The data collection instrument, based on a prior study,^10^ included data for identification (sex, age, weight, height, and skin color); socioeconomic status (profession, schooling level, family earnings, and residence); life habits (smoking, alcohol use, and physical activity); habit of measuring blood pressure; knowledge and beliefs regarding hypertension and treatment. In the statistical analysis, a significance level of p<0.05 was considered. The relation among the variables was evaluated by means of Fisher’s exact test.

## RESULTS

Practically half of the hypertensive patients were female, retired, and with elementary school level schooling. A little more than half reported not having white skin. An income of two to three minimum [monthly] wages predominated, and only 20% reported engaging in physical exercises. The mean age of the population analyzed was 64.1 years (SD=13.2), and the mean body mass index (BMI, kg/m^2^) was 35.9kg/m^2^ (SD=15.5). (Table 1).

**Table 1:** Biosocial characteristics and life habits of the hypertensive patients

Variables	n (%)
Sex	
Female	51 (51.0)
Male	49 (49.0)
Ethnicity	
White	44 (44.0)
Nonwhite	56 (56.0)
Schooling level	
Elementary	51 (51.0)
High School	24 (24.0)
College	14 (14.0)
Reads and writes/illiterate	9 (9.0)
Does not know	2 (2.0)
Occupation	
Works	32 (32.0)
Does not work	11 (11.0)
Retired	52 (52.0)
Unemployed	1 (1.0)
Housekeeper	4 (4.0)
Family [monthly] income, in minimum salaries*	
Up to 1	27 (27.0)
2-3	42 (42.0)
>3	22 (22.0)
Does not know/not informed	9 (9.0)
Smoker	13 (13.0)
Regular physical exercise	20 (20.0)
Ingestion of alcoholic beverages	38 (38.0)

*Minimum [monthly] salary: R$ 622.00.

The most often reported comorbidities were heart problems (acute myocardial infarct and heart failure) and diabetes; one fourth of the participants indicated having had a stroke. Most (59.0%) received orientation as to non-drug forms of treatment, the most frequent of which were losing weight and decreasing salt intake. It was noteworthy that 25% reported not using antihypertensive drugs, and 17.3% had not taken them in the previous 15 days (Table 2). The main reasons were forgetfulness (33%), the blood pressure “was OK” (29%), “I only take the medication when I feel ill” (12%), and undesirable effects (7%). About half said that they do follow-up with Primary Care, and we point out that one fourth of them reported no treatment for hypertension, besides those who had missed the medical visits in the previous year.

**Table 2:** Characteristics of the forms of drug and non-drug treatment and past history of the hypertensive patients

Variables	n (%)
Comorbidities (n=98)	
Heart problem	51 (52.1)
Diabetes	48 (49.0)
Hypercholesterolemia	43 (43.9)
Stroke	24 (24.5)
Orientation as to non-pharmacological treatment (n=97)	59 (60.8)
Orientations (n=59)	
Decrease salt intake	38 (64.0)
Lose weight	36 (61.0)
Engage in physical exercise	23 (39.0)
Using antihypertensive medications	75 (75.0)
Stopped taking medication	13 (17.3)
Frequency of visits to the physician	
Monthly	34 (34.0)
From 6 months to 1 year	34 (34.0)
Does not go	29 (29.0)
Goes when feels ill	3 (3.0)
Follow-up location	
Primary Care Unit	49 (49.0)
Hospital	36 (36.0)
Clinic	9 (9.0)
Not informed	6 (6.0)
Missed the medical visits in the previous year (n=94)	20 (21.3)

As to knowledge and beliefs, there was an expressive number of patients that believed that hypertension could not be cured; that the treatment should be extended for the rest of their lives; and that hypertension causes complications. Half of them indicated the highest grade for severity of the disease, and as to value for “high blood pressure,” the number of those who did not know was noteworthy (Table 3). The most often reported complications were stroke (52%), acute myocardial infarct (41%), and renal complications (15%).

**Table 3:** Beliefs and knowledge of the hypertensive patients

Variables	n (%)
Duration of treatment for hypertension	
Limited time	7 (7.0)
All their life	77 (77.0)
Did not know	16 (16.0)
Grade for severity of hypertension	
1-2	9 (9.0)
3-4	33 (33.0)
5	50 (50.0)
Did not know	8 (8.0)
High blood pressure causes complications	84 (84.0)
High blood pressure can be cured	25 (25.0)
Value of high blood pressure, mmHg	
<140/90	25 (25.0)
≥140/90	38 (38.0)
Does not know	37 (37.0)

Of the hypertensive patients that informed the value of their blood pressure, it was noted that 46.7% were not controlled. The lack of control was associated with non-white ethnicity and absence of heart problems (p<0.05) (Table 4).

**Table 4:** Controlled and non-controlled hypertensive patients and associated variables

	Hypertensive patients		Total	p value
	
	Controlled	Non-controlled		
	n (%)	n (%)	n (%)	
	(n=41)	(n=36)	(n=77)	
Ethnicity*				0.049
White	24 (58.5)	13 (36.1)	37 (48.1)	
Non-white	17 (41.5)	23 (63.9)	40 (51.9)	
Heart problem*				0.038
Yes	25 (62.5)	13 (37.1)	38 (50.7)	
No	15 (37.5)	22 (62.9)	37 (49.3)	

*Fisher’s exact test p≤0.05.

## DISCUSSION

The first *datum* that calls attention was that about two years after hospitalization, more than one third of the hypertensive patients died. Such an event may be justified by not following correctly the antihypertensive treatment, and consequently, by elevated pressure levels that can cause lesions in target organs. Low compliance with the treatments may be responsible for the inadequate control of the disease. Control of hypertension, despite being recommended by health policies,^11,12^ shows low rates, such as 45.5% in a study performed at a Primary Care setting^13^ and rates quite a bit lower in review studies.^5^ We point out that the lack of control was associated with non-white ethnicity, corroborating literature data,^14,15^ and the national survey, which showed that the risk of stroke was greater in black-skinned individuals, regardless of sex, and even considering hypertension as the basic cause of death.^6^ The lack of control was also associated with the absence of heart problems, probably considering greater compromise with health of hypertensive patients affected by heart problems.

We also add that unfavorable characteristics of hypertensive patients, such as low income, difficulties in access to healthcare services, and greater prevalence of risk factors, may have contributed not only to the low degree of control, but also to the death of hypertensive patients. Some comorbidities, such as diabetes, hypercholesterolemia, and past history of stroke reflect an aggravating profile of health conditions. Diabetes, the most frequent associated disease, appears as a significant cardiovascular risk factor, and when associated with hypertension, it is more deleterious.^16,17^

The adoption of healthy habits and life styles is an important tool in antihypertensive treatment of patients. Physical inactivity, obesity, and the prejudicial use of alcohol, added to smoking and hypercholesterolemia, are considered priority factors for intervention in hypertensive patients.^18-20^

We point out that knowledge and beliefs about hypertension are variables to be considered. Hypertensive patients were aware of complications caused by hypertension as reported in other studies,^21,22^ but most of them did not know which blood pressure value can be considered as hypertension. Such data indicate the scarce information of these patients as to important aspects related to a chronic disease. Other aggravation factors that might contribute to lack of control of the disease were identified, such as not complying with drug treatment, mentioned by one fourth of hypertensive patients, missing the medical visits, and having stopped taking the medications in the previous 15 days due to forgetfulness, “my pressure was OK”, and only using the drugs when they felt ill. The reasons contributing to low compliance with treatment, and consequently, to unsatisfactory levels of control are complex and varied. They include from aspects linked to the disease, as a consequence of chronicity and the absence of specific symptoms, to drug treatment, even treatment for the rest of one’s life, undesirable effects, and complex dosing schedules. As to non-drug treatment, to the changes in habits and life styles. As previously demonstrated,^2,8,10^ the use of the telephone contact tool proved effective, in order to allow the characterization and follow-up of hypertensive patients, promoting measures for improving treatment of this clientele.

The strategies proposed by healthcare professionals to modify the morbidity and mortality profiles of hypertensive patients are imperative, as well as the use of the telephone contact tool for follow-up and care of hypertensive patients. Among the limitations, we point out the descriptive and exploratory nature of the study - although important, it was not possible to establish a cause and effect relation.

## CONCLUSION

The present study showed the important mortality index after hospitalization and a multiplicity of factors that can compromise the adequate follow-up of hypertensive patients, mainly after an episode of hospitalization, which is often due to complications from lack of control of the disease.
